# The Importance of Cellular Immune Response to HIV: Implications for Antibody Production and Vaccine Design

**DOI:** 10.1089/dna.2021.0520

**Published:** 2022-01-12

**Authors:** Elena Brenna, Andrew J. McMichael

**Affiliations:** Nuffield Department of Clinical Medicine, University of Oxford, Oxford, United Kingdom.

**Keywords:** T follicular helper cells, HIV, vaccine platform, germinal centre, broadly neutralizing antibodies

## Abstract

Despite many years from the discovery of human immunodeficiency virus (HIV), a prophylactic vaccine against HIV is still needed. The failure of most of the vaccine clinical trials in the field has different causes, mainly due by the difficulties to identify the correct antigen able to prime the optimal B cell lineage and then make the series of somatic mutations necessary to generate broadly neutralizing antibodies (bNAbs). B cells are responsible for the bNAbs production; however, their function is strongly influenced by the presence of a population of CD4^+^ T lymphocytes, mainly present in the lymphoid organs, the T follicular helper cells (Tfh). In this review, the importance of the contribution of Tfh cells in HIV response is highlighted and future therapy perspectives based on these observations are described. The advanced technology available nowadays and the wide knowledge built over the past years for HIV may eventually create the best scenario for the generation of an effective vaccine.

## Introduction

The extraordinary complexity and precise organization of the immune system allows us to have an efficient tool against infectious agents and cellular damage. A central property of immunity that is fascinating is its capacity to react against foreign pathogens that have not been encountered previously. The immune system can therefore generate a specific response by delivering targeted effector functions to kill the foreign invader and at the same time self-regulating the response, to limit excessive activation. This is possible thanks to both the innate and the adaptive immune systems, two indissoluble processes that interact and collaborate with each other to enable an effective immune response (Mittrucker *et al.*, 2014; Sallusto, 2016; Samji and Khanna, 2017).

The sentence “there is no future without memory,” often used in other aspects of life, also applies to an immune response. Immunological memory, a cardinal feature of the adaptive immune system, ensures recall of immunity to a previous pathogen and therefore a rapid and highly effective response upon re-exposure. Both T and B cell lymphocytes, critical players of the adaptive immune system, are able to remember a pathogen for a long time and to generate either a cellular response and directly differentiate into a cytotoxic function, cytokine-mediated help for different elements of the immune system, and a humoral response mediated by secretion of antibodies (Hammarlund *et al.*, 2003; Lanzavecchia and Sallusto, 2009). The concept of immunological memory therefore allows the system to be ready, prepared, and more efficient during a second exposure. During an infectious response, two major populations of T lymphocytes play crucial roles: CD8^+^ T cells are fundamental to directly kill infected cells, whereas CD4^+^ T cells help CD8^+^ T cells and sustain the maturation of highly specific antibodies produced by B lymphocytes and therefore are essential elements for an effective immune response.

In the 20th century, the well-structured human immune system had to face the challenge of an incredibly evolved virus that has put the entire immunological mechanism to the test. With the human immunodeficiency virus (HIV), immunological memory is particularly attacked: in addition by specifically targeting the essential population of CD4^+^ T lymphocytes, the virus can tire the immune system by mutating its surface very rapidly and extensively within each infected person enabling immune escape (McMichael *et al.*, 2010; Liu *et al.*, 2013). Every mutation stimulates a different effector and a consequent memory response, until there is exhaustion of the entire immune machinery.

The power of HIV hence is to attack both the generation of effector and memory responses, which are the necessary elements for an efficient vaccine (McMichael, 2006; Haynes *et al.*, 2016).

Fighting HIV is challenging and exhausting for the whole immune system, leading to immunodepression in patients, who are also often affected by opportunistic infections. Robust and efficient cellular and humoral responses are necessary, although rarely sufficient to control the virus infection (Liu *et al.*, 2013; Ferrari *et al.*, 2017; Kervevan and Chakrabarti, 2021).

It is therefore not surprising that despite the 38 years from the identification of the virus, an effective vaccine against HIV still does not exist. The reasons of this lack are various, owing mainly to the difficulty in identifying a candidate antigen that can both prime B cells of an appropriate genetic lineage and also promote antibody affinity maturation and somatic hypermutation to generate antibodies that broadly neutralize different strains of the virus. Ideally this would prevent any immune cells becoming infected and thereby compromised (Haynes *et al.*, 2014).

The incidence of people infected by HIV in 2019 was still 1.7 million (https://www.who.int/teams/global-hiv-hepatitis-and-stis-programmes/hiv/strategic-information/hiv-data-and-statistics 2021). Although the quality of life in patients infected with HIV in the last 20 years has tremendously improved, much HIV research is still needed to improve the therapies available and their distribution and ultimately to eradicate the disease by developing a potent prophylactic vaccine.

## B Cells and Antibody Production in the HIV Context

Antibodies are fundamental for the clearance of most of the external pathogenic agents. Their main roles are not only to neutralize the pathogens but also to alert and recruit other elements of the immune system (e.g., complement, cytotoxic cells), which are directly involved in the killing of the pathogen (Birgitta, 2000; Posch *et al.*, 2020).

**FIG. 1.**
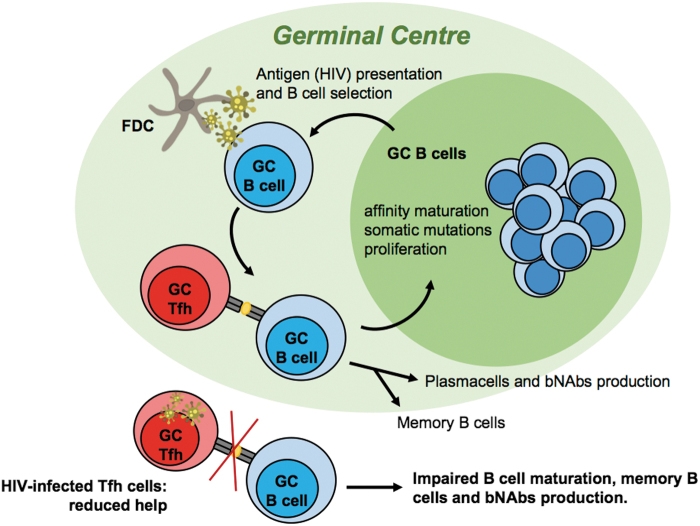
Schematic representation of the GC reaction in the B cell follicle during HIV infection. FDC, follicular dendritic cells; bNAb, broadly neutralizing antibodies; GC, germinal center; HIV, human immunodeficiency virus; Tfh, T follicular helper cells.

But how can our body generate effective and specific antibodies against something that we have never seen before? After an antigen challenge, dynamic temporary structures in the secondary lymphoid organs are formed by specialized immune cells: these structures are called germinal centers (GCs) (Fig. 1). Here B cells are chosen for generation of highly specific antibodies by encountering the cognate antigen several times, mainly held by follicular dendritic cells (FDCs), and undergoing rounds of somatic mutation and selection by antigen so as to refine their surface receptor (Victora and Nussenzweig, 2012; Bannard and Cyster, 2017). At the end of these dynamic cycles, B cells that are exiting the GC likely produce higher affinity antibodies specific for the pathogen in question. GC formation is highly energy intensive and requires effort from each element of the immune system. Each GC usually focuses on one particular antigen and can generate a limited number of antigen-specific B cells. B cells exit the GC can become either antibody-producing cells, also known as plasmablasts, or memory B cells (Mesin *et al.*, 2016).

In the context of HIV infection, every additional mutation of the virus envelope protein, which enables the virus to escape recognition of the immune cells, has the potential to generate a new GC, and a subsequent B cell maturation process. The production of neutralizing antibodies that for a common slowly evolving antigen takes only few weeks, for the highly mutating HIV Envelope requires several years (Kwong and Mascola, 2018).

Often when the system manages to generate neutralizing antibodies against the virus, the latter mutates requiring a restart of the GC process and the generation of new antibodies (Liu *et al.*, 2013). The consequence is often the exhaustion of the immune system, by making it impossible to control the infection. Besides the ongoing vicious cycle of antibody mutation leading to envelope mutation and escape, there are other reasons why it is difficult to generate an antibody response against HIV. One is the low number of Envelope molecules on the virus surface meaning that antibodies cannot take advantage of high avidity binding and can only bind as low affinity monomers (Layne *et al.*, 1992; Chertova *et al.*, 2002; Brandenberg *et al.*, 2015); the ability of the virus to generate a provirus reservoir where the virus is silent for years and not exposed to the immune system (Yang *et al.*, 2018). All these combine to resist immune responses.

## Role of T Follicular Helper Cells in HIV Response

B cells cannot do everything by themselves, they need good helpers. T follicular helper cells (Tfh) are specialized CD4^+^ lymphocytes mainly localized in the follicles, which are specific sites of a lymphoid organ where GC structures are generated (Crotty, 2019). Tfh cells have an essential and fundamental role in helping B cells to generate broadly neutralizing antibodies (bNAbs). Naive CD4^+^ T cells are primed by dendritic cells in the T cell zone of the secondary lymphoid organs and the acquisition of a specific phenotype, such as the expression of CXCR5 on their surface, allows Tfh-like cells to migrate to the B cell border where the cognate contact with B cells promotes their maturation in the GC reaction (Law *et al.*, 2020). Furthermore, in the GC selected antigen-specific Tfh cells provide help to the cognate B cells and most importantly decide the fate of B cells in the GC: B cells that are coming out from their somatic hypermutation process, contact antigen-specific Tfh cells, and present Envelope-derived peptides, bound to class II HLA molecules to the T cell receptors at the immunological synapse. This process enables the T cell to determine whether the B cell is ready to exit the GC and become plasmablast or memory cells, or whether it has to re-enter into the GC cycle to refine its antibody receptor further or whether it cannot survive any longer (Papa and Vinuesa, 2018). It is almost impossible to have efficient antibody production without an adequate Tfh response (Fig. 1).

CD4^+^ T cells with similar features have been found in the peripheral blood (Morita *et al.*, 2011), also known as circulating Tfh (cTfh), and recent studies showed the correlation between cTfh and the Tfh cells resident in the GC, in addition to their distinction with the rest of memory CD4^+^ T cells (Locci *et al.*, 2013; Brenna *et al.*, 2020). The Tfh response can therefore be monitored in the circulation and is a helpful parameter to consider when assessing the immunogenicity of a vaccine in a clinical trial.

Because Tfh cells are a subset of CD4^+^, they are one of the main targets of HIV and are infected at higher frequencies compared with non-Tfh cells. The high activation state of Tfh cells in the GC and their exposure to cells that present and process live virus (e.g., FDCs and B cells), makes them highly susceptible to HIV infection, limiting their functionality in acute and chronic immune responses (Moysi *et al.*, 2018; Xu *et al.*, 2019). Thus, HIV infection impairs the ability of Tfh to help B cells in the GC, contributing to the inadequate production of bNAb in >90% of patients (Simek *et al.*, 2009; Kumar *et al.*, 2018).

To support the importance of Tfh in viral response, studies have reported that individuals who develop bNAb against HIV have a higher number of antigen-specific cTfh cells with fewer regulatory controls (Locci *et al.*, 2013; Moody *et al.*, 2016). HIV can remain silent for years especially in patients treated with antiretroviral therapy (ART) and create a virus reservoir in cells that have been infected without producing new virus. It has been shown that Tfh cells are often the site of the virus reservoir, most probably because of their high infection rate (Aid *et al.*, 2018).

## Future Perspective: Tfh Cells as a Possible Target for Future HIV-1 Vaccines?

Extremely powerful therapies exist nowadays to control HIV-1 infection, based on the antiretroviral drugs that have been developed and improved in the last 20 years to reduce the virus infectivity, stop the virus replication, and therefore limit the virus aggressiveness. Nevertheless, antiretroviral drugs can cause virus resistance in many patients chronically infected, especially in countries where the drugs are still too expensive for the majority of the patients and the correct compliance and adherence are challenging (Lingappa, Lingappa *et al.*, 2021; Mbhele *et al.*, 2021).

A big effort has been made in HIV research to identify and isolate bNAbs generated by patients who managed to control the virus. Hundreds of antibodies have been isolated with the purpose of being delivered as therapeutic or prophylactic therapies. Most of them, already in an advanced stage of clinical trials, often in combination with ARTs, are showing promising results and may be soon introduced as therapy in certain clinic conditions (Sok and Burton, 2018; Haynes *et al.*, 2019; Liu *et al.*, 2020), but they are likely to be prohibitively expensive.

As described previously, Tfh cells play an important role in HIV response and recent therapeutic strategies are indeed attempting to boost Tfh cell function to obtain a more efficient vaccine.

A vaccine platform of mRNA encapsulated in lipid nanoparticles (mRNA-LNPs) has been recently characterized for several infectious diseases, in particular for SARS-CoV-2, and also for HIV. The power of this approach, besides the positive aspects of using mRNA in vaccine design, is the induction of both Tfh and B cells in the GC response. Current trials are ongoing in different animal models and data are encouraging for future human applications (Pardi *et al.*, 2018; Mu *et al.*, 2021). The effectiveness of mRNA-LNPs platforms, successfully demonstrated during the SARS-CoV2 pandemic, will likely help soon the containment of other infectious disease.

Another recent strategy for enhancing vaccine stimulation of bNAbs in HIV vaccine design is the development of an immunogen that consists of an HIV surface protein coupled to a highly immunogenic polypeptide such as a fragment of the tetanus toxoid (Pizza *et al.*, 2004; Ou *et al.*, 2020). This construct should boost the Tfh response in the context of HIV vaccination and facilitate the help of B cells to produce antibodies.

Both these examples are only two of the exciting ideas being developed to improve HIV vaccine design, particularly stimulated by the extraordinary rapid and successful advances in the response to the Covid-19 pandemic.

The basic and intense research in the last 30 years has been fundamental to understand the virus and the immune response to it. Now for the first time the technology available and the wide knowledge have the power and the possibility to build an effective vaccine against HIV.
